# Supplementary material for: Kinetics of initiating polypeptide elongation in an IRES-dependent system
Source: eLife. 2016 Jun 2;5:e13429. doi: 10.7554/eLife.13429 (PMC4963199; doi:10.7554/eLife.13429)
Supplement: Supplementary file 1. — DOI: http://dx.doi.org/10.7554/eLife.13429.016 [file elife-13429-supp1.docx]

| Table S1. Initial coding sequences of variants used in this work | |
| --- | --- |
| IRES | Coding sequence |
| Wt-IRES | GCU ACA UUU CAA GAU ACC AUG GAA |
| F-IRES | UUC ACA UUU CAA GAU ACC AUG GAA |
| FM-IRES | UUC AUG ACA UUU CAA GAU ACC AUG |
| FKM-IRES | UUC AAA AUG ACA UUU CAA GAU ACC |
| FVKM-IRES | UUC GUG AAA AUG ACA UUU CAA GAU |
| FKVRQWLM-IRES | UUC AAA GUG AGA CAA UGG CUA AUG |
